# Cytotoxic effect and tissue penetration of phenol for adjuvant treatment of giant cell tumours

**DOI:** 10.3892/ol.2013.1244

**Published:** 2013-03-11

**Authors:** FALK MITTAG, CARMEN LEICHTLE, INA KIECKBUSCH, HARTWIG WOLBURG, MAXIMILIAN RUDERT, TORSTEN KLUBA, ULF LEICHTLE

**Affiliations:** 1 Department of Orthopaedic Surgery, University Hospital Tuebingen, Tuebingen 72076;; 2 Institute of Pathology and Neuropathology, Department of General Pathology, University Hospital Tuebingen, Tuebingen 72076;; 3 Department of Orthopaedic Surgery, University Hospital Wuerzburg, Wuerzburg 97074, Germany

**Keywords:** phenol, tissue penetration, giant cell tumour, GCT, cytotoxic, denaturation

## Abstract

Local adjuvant treatment of giant cell tumours (GCTs) of the bone with phenol has led to a significant reduction in recurrence rates. In the current study, the optimal phenol concentration and duration of intralesional exposure were evaluated. Specimens of GCTs were exposed to various concentrations of phenol solution (6, 60 and 80%) for either 1 or 3 min. Following embedding in glutaraldehyde, the tumour cell layers were examined by transmission electron microscopy. Destroyed cell organelles indicated the penetration depth as a sign of denaturation. Incubation of GCT specimens with 6% phenol solution for 3 min resulted in the most tissue damage and the deepest tissue penetration of ∼200 *μ*m. Incubation with 60 and 80% phenol solution reached a penetration depth of only ∼100 *μ*m. Phenol instillation may be used for the treatment of small scattered cellular debris following intralesional curettage; however, it is not suitable for treatment of remaining solid tumour tissue of GCT. The use of high phenol concentrations has no benefit and increases the risk of local or systemic intoxication.

## Introduction

Giant cell tumours (GCTs) account for ∼8% of all bone tumours (1,2), commonly arising from the meta-epiphyseal region of the knee (condyles and tibial plateau), proximal humerus and distal radius (3,4). Wide resection of these tumours lowers the local recurrence rate but often results in a loss of function due to extensive joint resection (2,3). Therefore, extended intralesional curettage has become the recommended treatment (5,6). Combined with various adjuvant therapies, including phenolisation, ethanolisation, rinsing with H_2_O_2_, heat (electric cauterisation or cementation) cryosurgery, burring and argon beam coagulation, recurrence rates vary from 5–50% (7–12). Among the additional adjuvant therapies, phenolisation (chemical cauterisation) and cementation (thermic cauterisation and stabilisation of the bone defect) are common treatments (8,11,13–15). After curettage of the tumour a phenol solution is instilled and should cover the whole cavity. Phenol is highly toxic and supposed to eliminate the majority of the remaining tumour cells by denaturation (16). In this context, little is known concerning the necessary concentration, the duration of exposure and the depth of tissue penetration of the phenol solution. Commonly used phenol concentrations for the treatment of GCT are either low (5–6%) or very high (60–80%) (8,14,15). Therefore, we exposed GCT specimens to various concentrations of phenol. The time-dependent depth of tissue penetration and denaturation of cells were evaluated using transmission electron microscopy.

## Materials and methods

### Samples

Histologically determined GCTs of 3 patients (2 proximal tibia and 1 metatarsal bone) were surgically removed at the Department of Orthopaedic Surgery, Tuebingen, Germany. Additionally, 6% phenol instillation and cementation were performed. Viable solid tumour tissue specimens (∼0.5 cm in diameter) of the removed GCTs were obtained and tested *in vitro*. All patients provided informed consent to partake in the study. The study was approved by the local ethics committee (Nr. 605/2011BO2).

### Preparation of specimens

Phenol solution (6, 60 or 80%) was added to the surface of the tumour specimens for either 1 or 3 min *in vitro*. Following washing with 0.9% NaCl solution, specimens were immediately embedded in paraffin, sliced and stained. In addition, following phenolisation, each specimen was examined by transmission electron microscopy. Briefly, tissues were fixed with 2.5% glutaraldehyde (Paesel and Lorei; Frankfurt, Germany) buffered in 0.1 mol/l cacodylate buffer (pH 7.4). Thereafter, the tissues were postfixed in the same fixative as used previously for an additional 4 h, then post-fixed in 1% OsO_4_ in 0.1 mol/l cacodylate buffer and dehydrated in an ethanol series (50, 70, 96 and 100%). The 70% ethanol solution was saturated with uranyl acetate for contrast enhancement. Dehydration was completed in propylene oxide. The specimens were embedded in Araldite (Serva; Heidelberg, Germany). Semi- and ultra-thin sections were produced on an FCR Reichert Ultracut ultramicrotome (Leica, Bensheim, Germany). The semi-thin sections were stained with toluidine blue for inspection, while the ultra-thin sections were mounted on pioloform-coated copper grids, contrasted with lead citrate, and analysed and documented with an EM10A electron microscope (Carl Zeiss; Oberkochen, Germany). The penetration depth of phenol was observed to be dependent on the destruction of cell organelles in the deeper cell layers.

## Results

**Figure 1:**
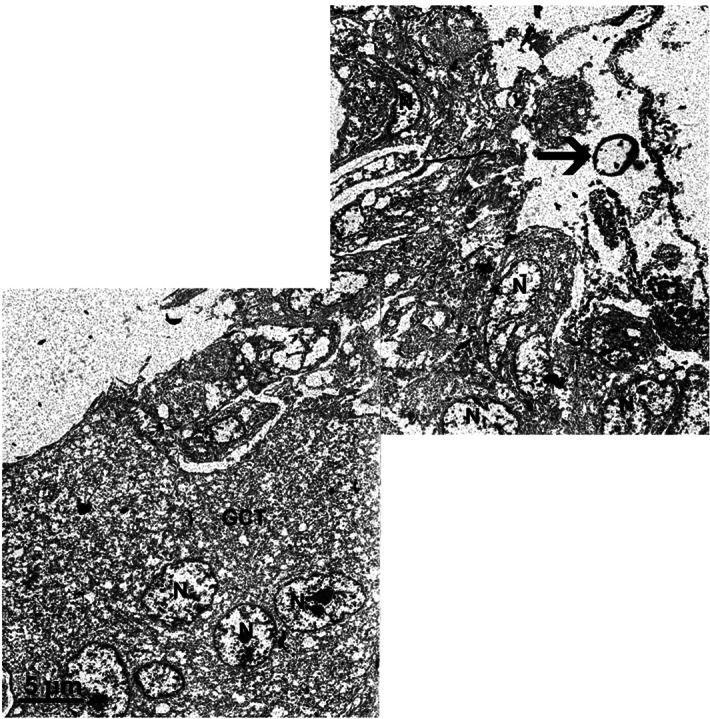
Electron microscopical analysis of the treatment of a giant cell tumour (GCT) with 6% phenol for 1 min, directly at the surface of the probe. The arrow indicates a cell that was completely coagulated; however, this zone of destruction remains rather small. Only 10–15 *μ*m from the phenol diffusion front, the cellular structure is observed to be normal. A cell of the GCT with several nuclei (N) is shown.

**Figure 2:**
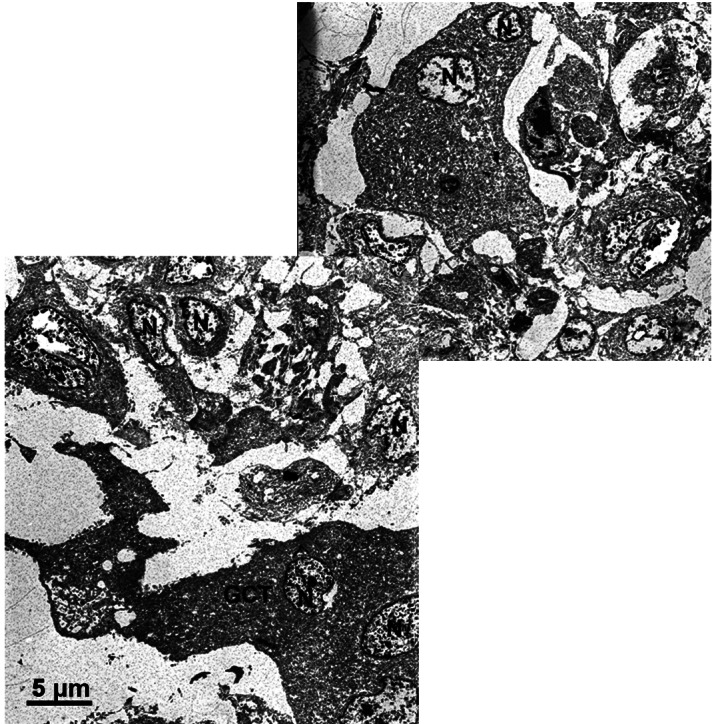
Electron microscopical analysis of the treatment of a giant cell tumour (GCT) with 6% phenol for 1 min, ∼10 *μ*m along from the zone shown in Fig. 1. As already evident in the left panel of Fig. 1, the cellular structure is relatively well maintained.

**Figure 3:**
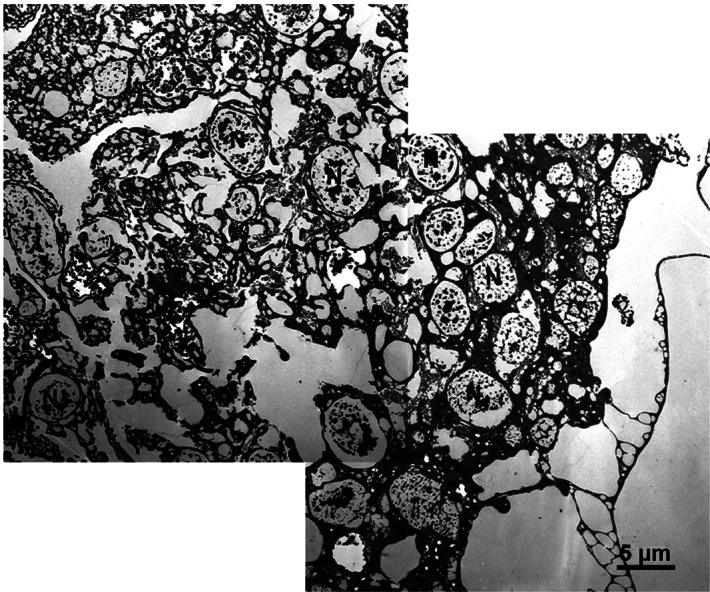
Electron microscopical analysis of the treatment of a giant cell tumour (GCT) with 80% phenol for 3 min, directly at the surface of the probe. In contrast to the lower phenol concentrations and lower treatment time, all cells are completely coagulated. The cells are visualised only as scaffolds of coagulated proteins, and no longer as complex structures of cyto- and nucleoplasms enclosed by cell membranes or nuclear envelopes, respectively. Therefore, this type of cell death cannot be categorised as necrosis or apoptosis, as these processes both precede reactive or even active mechanisms as a response to toxic agents of different types. In this case, an immediate passive coagulation of cells treated with phenol and not characterised by a specific biological reactivity is observed.

Incubation with 6% phenol solution for 1 min resulted in a damage to only the uppermost cell layers (10–20 *μ*m; Figs. 1 and 2). Damage was represented as a complete coagulation of the cytoplasm and in particular the nucleoplasm. No complete loss of any of the cell substructures was observed. The outlines of the organelles were simply converted to black. After 3 min, the penetration depth increased to ∼200 *μ*m. Incubation with the 60 and 80% phenol solution for 1 min resulted in the destruction of 10 cell layers and a penetration depth of ∼100 *μ*m. After 3 min of 60 and 80% phenol exposure, neither additional tissue damage or an increase in the penetration depth were observed (Fig. 3 and Table I).

## Discussion

The greatest penetration depth and tissue destruction in GCT were observed when 6% phenol solution and a contact time of ≥3 min were employed. This type of destruction cannot be described as cell death in the sense of necrosis or apoptosis, as such types of cellular destruction imply a reaction (necrosis) or an active execution (apoptosis) as a consequence of toxic treatment. Higher concentrations of phenol led to extreme destruction of the uppermost cell layers, but not to a deeper infiltration of the tissue. Superficial denaturised tissues were considered to act as a barrier preventing further penetration of phenol.

Due to the complex investigation of each single cell layer with transmission electron microscopy and the achievement of consistent results in all three tumours, we did not extend the study. Nevertheless, it is possible that a deeper tissue penetration depth may be achieved by 6% (or a slightly higher concentrated) phenol solution with an incubation time >3 min. This requires further investigation.

The penetration depth of various concentrations of phenol in GCT has not yet been investigated. According to the literature, phenol concentrations used in the treatment of GCTs vary from 5–75% (8,14,15). The incubation time is often not mentioned. Quint *et al* investigated the cytotoxic effect of different phenol concentrations on single-layer sarcoma cell lines and recommend the use of a phenol concentration of 5% (17). Evaluation of the penetration depth was not possible with this setting. Lack *et al* investigated the denaturising effect of a 75% phenol solution on normal tissue, tumours and chondromatous tissue, using light microscopy (18). The penetration depth in soft tissue varied from 40–500 *μ*m. In chondromatous tissue, no cytotoxic effect was evaluated. This suggests that phenol has no toxic effect on bone.

High concentrations of phenol are capable of causing local chemical burs; contact of the healthy surrounding tissue with the solution ought to be avoided. Phenol is locally absorbed and excreted in the urine. In this regard, Quint *et al* described a low risk for humans depending on the quantity of 5% phenol solution used (16).

The results of the present study suggest that an instillation of 6% phenol solution for ≥3 min is the most effective method for denaturising as many of the remaining tumour cells as possible. High phenol concentrations did not demonstrate a benefit, and they increased the risk of bone necrosis and systemic intoxication. The determined optimal phenol concentration and incubation time for GCT are not transferable to the treatment of other tumours.

Phenol instillation (6% for ≥3 min) may be used for the denaturation of small, scattered, cellular debris following intralesional curettage of GCT; however, due to the relatively low tissue penetration of 200 *μ*m, it is not suitable for treatment of the remaining solid tumour tissue. Adequate surgical removal of the tumour remains to be the most important predictive factor in preventing recurrence of GCT of the bone.

## Figures and Tables

**Table I:** Penetration depth of phenol in GCT specimens incubated with different concentrations of phenol soution.

		Penetration depth (*μ*m)		
Phenol concentration (%)	Exposure time (min)	Specimen 1	Specimen 2	Specimen 3
6	1	15	10	25
6	3	180–200	No data	200
60	1	160	80	100
60	3	80	100	No data
80	1	80	80–90	60
80	3	80–100	100	100

GCT, giant cell tumour; incubation with 6% phenol solution for 3 min resulted in the deepest tissue penetration.
